# The evolution of imprinting in plants: beyond the seed

**DOI:** 10.1007/s00497-021-00410-7

**Published:** 2021-04-29

**Authors:** Sean A. Montgomery, Frédéric Berger

**Affiliations:** Gregor Mendel Institute (GMI), Austrian Academy of Sciences, Vienna BioCenter (VBC), Dr. Bohr Gasse 3, 1030 Vienna, Austria

**Keywords:** Imprinting, Evolution, Plants, Embryo

## Abstract

Genomic imprinting results in the biased expression of alleles depending on if the allele was inherited from the mother or the father. Despite the prevalence of sexual reproduction across eukaryotes, imprinting is only found in placental mammals, flowering plants, and some insects, suggesting independent evolutionary origins. Numerous hypotheses have been proposed to explain the selective pressures that favour the innovation of imprinted gene expression and each differs in their experimental support and predictions. Due to the lack of investigation of imprinting in land plants, other than angiosperms with triploid endosperm, we do not know whether imprinting occurs in species lacking endosperm and with embryos developing on maternal plants. Here, we discuss the potential for uncovering additional examples of imprinting in land plants and how these observations may provide additional support for one or more existing imprinting hypotheses.

## Introduction

The term imprinting was coined by Helen Crouse in 1960 who described a process of parent-of-origin specific chromosome elimination during sex determination in black flies (Sciara), happening in both the soma and the germline, and differing between males and females (Crouse 1960). This led to the hypothesis that chromosomes carried a mark, an imprint, of their parental origin, which is carried across cell divisions. Since then, parental genomic imprinting has been discovered and studied in detail in placental mammals and flowering plants where it affects single genes and gene clusters, as opposed to the whole chromosomes of Sciara (Kelsey and Feil 2013; McGrath and Solter 1984; Surani et al. 1984). One exception is the imprinting of the X chromosome, wherein the paternal X chromosome is preferentially inactivated in specific embryonic (Okamoto et al. 2004), extraembryonic (Takagi and Sasaki 1975) and somatic cells (Deakin 2013) of particular mammalian species. The modern definition of imprinting encompasses its molecular phenotype, that is an epigenetic phenomenon in which alleles are expressed in a parent-of-origin specific manner. An epigenetic mark, or “imprint”, is established prior to fertilization that serves to direct the asymmetric silencing of alleles. Most imprinted genes are marked by DNA methylation (Batista and Köhler 2020), though studies in plants (Jullien et al. 2006; Moreno-Romero et al. 2019) and more recently in mouse (Chen et al. 2019; Inoue et al. 2018) have highlighted a role for the repressive histone modification H3K27me3.

While imprinting may potentially occur in all sexually reproducing organisms, it has only been described in placental mammals, flowering plants and some insect species. From such a sparse distribution it follows that imprinting must have arisen through convergent evolution, and thus raises the question about the selective pressures that favour the evolution of imprinting. A consensus on why imprinting has evolved remains elusive. Given the array of examples that support one or more hypotheses, it is likely that imprinting may arise under different selective pressures. In seed plants, evolutionary selection applies to the fitness of the offspring in terms of seed-proper development, maturation, survival, germination and survival of the seedling, whereas in non-seed plant offspring fitness is primarily a function of embryo survival and spore production.

The hypothesis that has arguably gained the most traction is the parental conflict hypothesis, also known as the kinship theory (Haig and Westoby 1989). This hypothesis posits that genomic imprinting is evolutionarily favoured when the interests of parental alleles in offspring differ from each other, resulting in the expression of whichever allele is favoured to be expressed (Haig 2014). Coincidently, the hypothesis was originally formulated to cover maternal investment in offspring in flowering plants (Haig and Westoby 1989). The differential dosage hypothesis extends the parental conflict hypothesis to a broader range of parental interactions that lead to the differential expression of parental alleles, rather than binary on or off states (Dilkes and Comai 2004). Nonetheless, both hypotheses deal with the same selective pressure, that is contrasting optima in gene expression levels between maternal and paternal alleles in offspring. Thus, the parental conflict and differential dosage hypotheses will not be distinguished further in this review.

Another prominent hypothesis relevant to imprinting in land plants is the coadaptation hypothesis (Wolf and Hager 2006). The coadaptation hypothesis focuses on maternally expressed imprinted genes and proposes that these alleles are preferentially expressed because it allows for improved coordination of resource transfer and growth between mother and offspring across a range of phenotypes (Wolf and Hager 2006).

It has been hypothesized that imprinting functions as a post-zygotic barrier in polyploids due to incompatibilities in gene expression levels of imprinted genes in interploidy crosses (Schatlowski and Köhler 2012). However, this hypothesis relies on pre-existing imprinting mechanisms that act as a reproductive barrier. It does not deal with the evolutionary origins of imprinting and will not be discussed here. Likewise, the hypothesis that individual imprinted genes have arisen under weak or relaxed selection (Berger et al. 2012; Rodrigues and Zilberman 2015) relies on the pre-existence of imprinting mechanisms that inadvertently act on these genes. This idea does not address the origin of imprinting and will also not be discussed here.

An excellent overview of these hypotheses is covered by recent reviews (Patten et al. 2014; Rodrigues and Zilberman 2015), as is a general overview of the evolution of imprinting in animals and plants (Sazhenova and Lebedev 2021). In this review, we examine the observations and theories surrounding the evolution of imprinting in land plants and the predictions resulting from them regarding the prevalence of imprinting in non-angiosperm species.

## Imprinting in land plants: a spotlight on angiosperms

Numerous reviews on imprinting in flowering plants comprehensively cover the topic (Armenta-Medina and Gillmor 2019; Batista and Köhler 2020; Gehring and Satyaki 2017) and we will briefly cover the basics here for the purpose of making comparisons to non-angiosperm species.

**Fig. 1:**
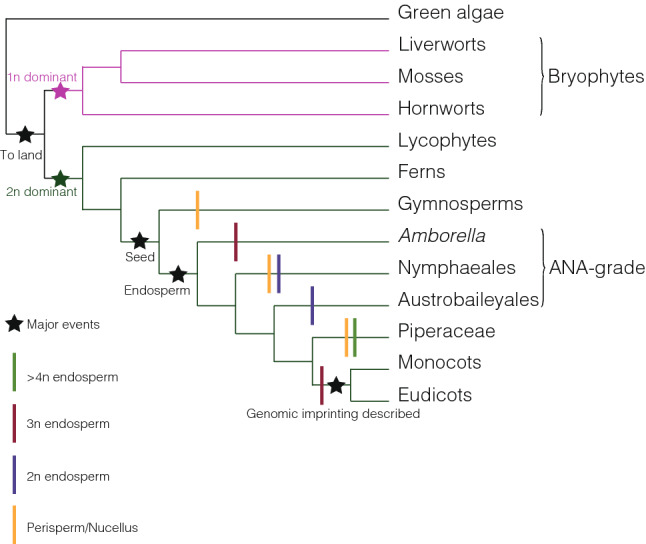
Schematic of land plant evolution. Schematic of major land plant groups and innovations relevant to imprinting. Major events are denoted with stars, including the terrestrialization of plants, dominance of haploid or diploid stages of the life cycle (also denoted in magenta and green), endosperm tissue resulting from a second fertilization event and where imprinting has thus far been described. Ploidy levels of endosperm and the presence of maternally derived resource storage tissues, the nucellus or perisperm, are also indicated

In flowering plants, seeds are the product of two fertilization events. The pollen tube delivers two sperm cells that fertilize the egg and the central cell. The fertilized egg develops as the embryo, while the fertilized central cell develops as the endosperm. The endosperm is usually triploid, directs the flow of nutrients from mother to embryo, and is surrounded by diploid tissues of maternal origin that differentiate from the ovule integuments. Amongst land plants, the search for imprinted genes has only been pursued in monocots and eudicots (Fig. 1). Of those genes identified, the vast majority are expressed in the endosperm (Gehring et al. 2011; Hsieh et al. 2011; Luo et al. 2011; Waters et al. 2011). There are around one hundred imprinted genes in maize and Arabidopsis, found in roughly equal proportions from both maternal and paternal genomes (Schon and Nodine 2017; Wyder et al. 2019). While some genes have been found to be imprinted across species and have strong effects on endosperm function when their imprinting is perturbed (Grossniklaus et al. 1998; Ingouff et al. 2005; Makarevich et al. 2008), there has been a wide debate regarding the conservation of the imprinted status of genes (Chen et al. 2018; Hatorangan et al. 2016; Klosinska et al. 2016; Lafon-Placette et al. 2018; Liu et al. 2021; Pignatta et al. 2014; Rong et al. 2021; Roth et al. 2018; Tuteja et al. 2019; Waters et al. 2013; Yang et al. 2018, 2020; Yoshida et al. 2018). Yet, a conservation of imprinting targets may exist for molecular complexes or pathways rather than individual genes. Difficulties in comparing the distinct modes of endosperm development amongst angiosperms also hinders establishing the degree of conservation (Kordyum and Mosyakin 2020). Regardless of their imprinted status, many imprinted genes have not been connected to obvious phenotypes when knocked out or when their imprinting is removed (Berger et al. 2012; Waters et al. 2013). The assessment of function might be precluded by redundancy and the lack of in-depth studies.

In contrast to the endosperm, only a small number of genes from both maternal and paternal genomes appear to be imprinted and expressed immediately following fertilization in the embryo (Jahnke and Scholten 2009; Nodine and Bartel 2012; Raissig et al. 2013; Zhao et al. 2019). Since the endosperm assumes the role of nutrient transfer and storage in the seed and often serves as the main interface between mother and embryo, imprinting in the embryo may have been attenuated or disappeared. However, the few imprinted genes identified in angiosperm embryos may be a remnant of more prevalent imprinting in the embryos of ancestral angiosperms.

Like the endosperm, the suspensor is a non-embryonic, transient tissue involved in nutrient transfer during early embryogenesis. There are reports of parent-of-origin effects on suspensor development (Bayer et al. 2009; Luo et al. 2016; Ueda et al. 2017; Zhang et al. 2017), and an analysis of parent-of-origin expression of suspensor genes has shown several hundred genes with biased expression throughout suspensor development (Zhao et al. 2020). Given that the endosperm is an angiosperm-specific tissue, it is conceivable that imprinting of genes in the suspensor or embryo is more likely to be conserved in species lacking endosperm, if imprinting were to be identified in those species.

Mechanistically, both H3K27me3 and DNA methylation are associated with imprinted genes, as observed in mammals. In angiosperms, H3K27me3 predominantly marks maternally imprinted alleles of paternally expressed genes, whereas DNA methylation predominantly marks paternally imprinted alleles of maternally expressed genes (Armenta-Medina and Gillmor 2019; Batista and Köhler 2020; Gehring and Satyaki 2017). Imprinted gene expression in endosperm is a result of the maintenance of an epigenetic asymmetry between parental alleles which has already been established in gametes. H3K27me3 is almost completely lost in sperm (Borg et al. 2020, 2021) and likely maintained in female gametes (Pillot et al. 2010), whereas DNA methylation is highly reduced in the female gametes (Jullien Pauline et al., 2012) and maintained in sperm (Calarco Joseph et al. 2012; Kawashima and Berger 2014). Therefore, an early clue to detect imprinting in non-angiosperm species may be the presence of an epigenetic asymmetry of H3K27me3 or DNA methylation in the gametes.

## Getting to the origins of imprinting and endosperm: ANA-grade angiosperms

The tight association of imprinting with endosperm in monocots and eudicots raises the question of whether imprinting in land plants is dependent on the existence of endosperm, and if so, whether there is a dependence on triploidy in endosperm. This last point is already questioned by the fact that endosperm ploidy is distinct from the triploid ratio of one paternal to two maternal genomes in some species of monocots and eudicots (Kordyum and Mosyakin 2020). Imprinting has not been investigated in land plants outside of monocots and eudicots, but endosperm can be found in ANA-grade angiosperms (Fig. 1). Several observations, mostly from interploidy crosses, indicate imprinting may be found in these species.

**Fig. 2:**
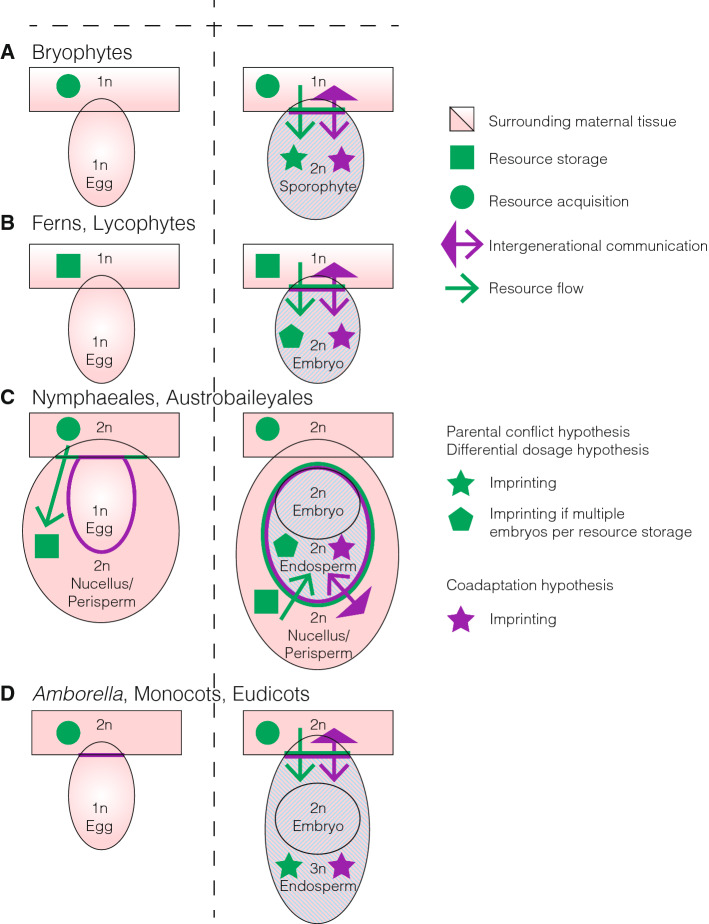
Embryonic development and intergenerational communication across land plants. Schematic of pre- (left) and post- (right) fertilization tissues relevant for imprinting in **a** bryophytes, **b** ferns and lycophytes, **c** Nymphaeales and Austrobaileyales and **d** *Amborella*, monocots and eudicots. Ploidy levels and tissue names are indicated inside the relevant tissues. Pink shapes indicate maternally derived tissues. Green circles indicate tissues where resource acquisition occurs, green boxes indicate tissues where resources are stored, and green lines indicate tissue boundaries across which resources are transferred. Purple arrows illustrate potential axes of communication between generations, with filled arrows denoting unfertilized maternal tissues and open arrows denoting post-fertilization tissues. Purple lines around tissues show the boundary between tissues from different generations. Stars indicate tissue in which imprinted genes are predicted to be found, whereas pentagons indicate imprinting is predicted if multiple embryos have access to the same resource storage tissue

In the Nymphaeales, interploidy crosses revealed contrasting parent-of-origin phenotypes. Extra paternal genomes cause increased endosperm growth, whereas extra maternal genomes cause decreased endosperm growth (Friedman et al. 2012; Povilus et al. 2018). These results mirror those in other angiosperms (von Wangenheim and Peterson 2004) and suggest the presence of imprinted genes in the endosperm in the Nymphaeales. It is interesting to note that the endosperm is diploid in the Nymphaeales (Geeta 2003; Floyd and Friedman, 2000), but the main resource storage tissue is the perisperm which derives entirely from the mother plant and develops prior to fertilization (Friedman 2008) (Fig. 2). Similarly, the Piperaceae and Austrobaileyales utilize a maternally derived perisperm or nucellus to store nutrients for the developing embryo (Losada et al. 2017; Madrid and Friedman 2010; Tobe et al. 2007), while the Piperaceae have a highly reduced polyploid endosperm (Madrid and Friedman 2010) and the Austrobaileyales have a large diploid endosperm (Losada et al. 2017; Tobe et al. 2007).

These observations are interesting, but in the absence of a clear demonstration of a parent-of-origin bias in the expression of specific genes, it remains unclear whether the observations reported above would challenge the importance of endosperm triploidy in the evolution and function of imprinting in the endosperm (Baroux et al. 2002; Stewart-Cox et al. 2004). *Amborella* has a triploid endosperm that is hypothesized to have originated independently from the triploid endosperm of monocots and eudicots, and this species provides the means to test for the relationship between a triploid endosperm and imprinting. Finding imprinted genes in the endosperm of *Amborella*, but not in the endosperm of Nymphaeales nor Austrobaileyales, would point to a strong connection between triploid endosperm and imprinting. In contrast, the presence of imprinted genes in the endosperm of all angiosperm groups would not support the correlation between imprinting and ploidy levels in endosperm. In conclusion, a high degree of variability of endosperm development in ANA-grade angiosperms may prove to be fertile ground to examine to what degree the evolution of imprinting is directly connected to the evolution of double-fertilization.

## Imprinting without seeds: observations from the past

We have so far covered imprinting in seed plants and would like to now consider what little is known about imprinting in the embryos of seedless land plants. An intriguing report in the aquatic fern *Marsilea vestita* describes a non-random segregation of paternal autosomes during embryonic mitoses after the 16-cell stage and suggests that an imprinting mark may underlie this unusual phenomenon (Tourte et al. 1980). Specifically, paternal chromosomes were labelled prior to fertilization and the label accumulated only in cells that will give rise to all aerial organs, whereas the label of maternal chromosomes was observed evenly throughout the fern embryo (Tourte et al. 1980). Similar results were later obtained in another aquatic fern, *Marsilea quadrifolia* (Bordonneau and Tourte 1994). If imprinting is behind these observations, this form of imprinting would more closely resemble imprinting in insects such as Sciara, where whole paternal chromosomes are imprinted (Crouse 1960). However, in the case of *Marsilea*, it is unclear whether paternal chromosomes are heterochromatinized, as in Sciara (de la Filia et al. 2021). This form of imprinting is also distinct from the more thoroughly studied imprinting of specific maternal and paternal loci in flowering plants and mammals. Since ferns lack sex chromosomes, this type of imprinting would also be distinct from the imprinting of the mammalian X chromosome. Whether and when a potential parentally biased expression would take place during embryogenesis in the model fern *Marsilea* remains to be tested by transcriptomic analyses. Likewise, neither immunostaining nor chromatin profiling experiments have been performed to identify an imprinting chromatin mark that may distinguish parental chromosomes, nor is the prevalence of this phenomenon across fern species known.

## Imprinting in bryophytes: whispers on the wind

We now turn our attention to the land plant groups comprising mosses, liverworts and hornworts, collectively referred to as bryophytes (Fig. 1). Like all land plants, bryophytes exhibit an alternation of multicellular haploid and diploid stages during the life cycle. However, in contrast to all vascular plants, the life cycle of bryophytes is characterized by the dominance of the haploid gametophytic stage (Shimamura 2015), rendering it the main stage for resource acquisition and support for the diploid embryonic sporophyte which remains attached to the maternal plant for the entirety of its development. The possibility of imprinting in bryophytes has been considered in detail (Haig 2013; Haig and Wilczek 2006), though no evidence has yet supported its existence.

From a theoretical standpoint, parental genomic imprinting is anticipated to take place in bryophytes (Carey et al. 2020b; Shaw et al. 2011). Several observations of bryophyte sporophytic development, mentioned below, suggest that imprinting may be found in these species. The direct and persistent interface between haploid mother and diploid offspring throughout the entirety of the life of the latter allows for prolonged crosstalk between the two. Extensive cell wall ingrowths and a unique cell wall composition in the region connecting the sporophyte to the maternal gametophyte are suggestive of a specialization to enable communication between the sporophyte and gametophyte (Moody 2020; Regmi et al. 2017). There is also the possibility for multiple embryos to develop per female gametophyte which can be sired by multiple males (Szovenyi et al. 2009). It has been hypothesized that the elongated seta, the stalk that connects the sporophyte to the gametophyte and elevates the former into the air, as well as stomata of moss sporophytes are innovations promoting resource transfer from gametophytes to sporophytes (Haig 2013).

Ultimately, the presence of imprinting in mosses will have to be determined by a detailed examination of crosses between distinct accessions with specific polymorphisms enabling parent-of-origin transcriptome analyses. In the moss Sphagnum, a preliminary analysis of sporophyte transcriptomes suggests parent-of-origin effects on transcription due to differences in gene expression between embryos borne on different maternal plants, but the authors concluded that these effects may be due to epigenetic, genetic, or maternal environmental effects (Shaw et al. 2016). The authors propose that more detailed analyses of these data, with the ability to discriminate the parental origin of transcripts, may provide valuable insights into imprinting and epigenetic effects on gene expression in moss sporophytes. Despite this initial report, an investigation into parent-of-origin biases of gene expression has not been conducted, thus imprinting has not been demonstrated in bryophytes.

If imprinting were to exist in bryophytes, recent results suggest that DNA methylation may be involved. In the liverwort *Marchantia polymorpha*, levels of DNA methylation in sperm are higher than in eggs and other tissues (Schmid et al. 2018). Mechanistically, this asymmetry of DNA methylation would allow for parental alleles to be distinguished from each other, and maintenance of this asymmetry on promoter regions may lead to the selective silencing of one allele. However, there is a lack of a direct report of DNA methylation on parental alleles during sporophyte development to reach a conclusion.

## Predictions of imprinting: gazing into the crystal ball

Under each hypothesis that attempts to explain the evolutionary conditions to allow for or favour imprinting, predictions can and have been made regarding its effects (Haig 2013; Patten et al. 2014; Rodrigues and Zilberman 2015). Here, we will briefly expand on these predictions, particularly in non-seed plants.

### Parental conflict and differential dosage hypotheses

Both the parental conflict hypothesis and differential dosage hypothesis revolve around contrasting optima in gene expression between maternal and paternal alleles in offspring (Dilkes and Comai 2004; Haig and Westoby 1989). Thus, imprinting would be predicted to occur when parental alleles in offspring would “disagree” on the level of expression. We will primarily illustrate this in examples of resource allocation from the mother plant to offspring, and consequently focus on tissues and timepoints in which resource transfer occurs.

In flowering plants, the endosperm is the primary post-fertilization tissue that fulfils the role of nutrient acquisition from and interfacing with the mother plant (Fig. 2D). The majority of imprinted genes are imprinted and expressed in the endosperm in monocots and eudicots, thus the endosperm appears to be the focus of imprinting in ANA-grade angiosperms under the parental conflict hypothesis. Parent-of-origin effects on endosperm growth in interploidy crosses indicate the presence of imprinted genes in this tissue and is supportive of the prediction under the parental conflict hypothesis that expressed paternal alleles of imprinted genes will favour increased endosperm growth, while the opposite is predicted for expressed maternal alleles. However, the relegation of resource storage to maternal tissues, the perisperm or nucellus, in the Nymphaeales and Austrobaileyales (Fig. 2C) may have resulted in a relaxation of selection for imprinted genes in the endosperm, as the pool of resources offspring may draw from is determined prior to fertilization by the maternal plant. One may consider the innovation of a maternal perisperm as an alternative strategy to the triploid endosperm with an extra copy of the maternal genome in eudicots and monocots, as both accomplish greater maternal control of resource allocation to offspring. *Amborella* lacks both a perisperm and nucellus and has a triploid endosperm that is thought to have originated independently from the triploid endosperm of monocots and eudicots (Fig. 1). Therefore, the same selective pressures should be acting on *Amborella* as other species in which imprinting has been described, and we would expect similar genes or pathways to be imprinted.

While genomic imprinting may exist in ferns, it is unlikely to support the parental conflict hypothesis. In most ferns, the main photosynthetic stage is the sporophyte, and the gametophytes are short-lived and therefore mostly function as a platform for fertilization. The gametophytes are not continuously nutritionally supported by the sporophyte and the resources invested into the gametophyte are determined prior to fertilization, similar in fashion to resource allocation to the perisperm in some ANA-grade angiosperms. In addition, most fern female gametophytes give rise to a single zygote and sporophyte (Fig. 2B), thus all resources can be dedicated to this single fertilization event and there is limited potential for future offspring and alternative uses for stored resources. Therefore, it can be envisaged that maternal and paternal alleles in the offspring would both be selected to maximize resource transfer from the gametophytic mother to the sporophytic offspring. However, some ferns have been observed to bear multiple embryos per gametophyte (Stone 1958). In this situation, maternal alleles in the offspring may be favoured to limit resource acquisition from the maternal gametophyte that could be used to nourish other offspring on the same plant, whereas paternal alleles may still be favoured to maximize resource acquisition. Therefore, comparing the presence or absence of imprinting between these two scenarios would clearly delineate support for or against the parental conflict hypothesis. Fern embryos are easier to access than those of other land plant species due to the relative dearth of encapsulating maternal tissue (Bell 1975), which facilitates investigations into the presence of imprinted genes and biased chromosome segregation in ferns.

Lycophytes have a similar life cycle structure as ferns, therefore the same predictions as above apply, though no report addressing imprinting in lycophytes has been found. Yet, one difference is that fertilization of lycophytes may take place on the maternal sporophyte inside the wall of the megaspore (Schulz et al. 2010; Spencer et al. 2020). While this type of maternal protection and investment may favour the evolution of imprinting, this type of fertilization occurs when the plants are self-fertilizing, thus “maternal” and “paternal” alleles are not distinguished as they both originate from the same individual.

A recent study using single-cell transcriptomics in Arabidopsis endosperm showed that, compared to the average in endosperm, a greater proportion of genes show imprinted expression in the chalazal endosperm, a specialized structure in the endosperm that directly interfaces with the maternal sporophyte (Picard et al. 2020). In bryophytes, the foot of the sporophyte is the tissue analogous to the chalazal endosperm, specialized for nutrient transfer between the maternal gametophyte and sporophyte (Shimamura 2015). Thus, the sporophyte foot may be a hotspot for imprinting. Additionally, the sporophyte remains connected to the gametophyte for the duration of its growth. Rapid growth may cause the sporophyte to act as a nutrient sink and thus function to draw resources from the mother plant since (Fig. 2A). Specific innovations such as continuous stomatal opening (Kubásek et al. 2021; Merced and Renzaglia 2013) and elongation of the seta (Haig 2013) may be controlled by imprinted genes and would warrant special consideration.

### Coadaptation hypothesis

An alternative to the parental conflict hypothesis, the coadaptation hypothesis, is not based on competing influences of the parental genomes in the offspring. Instead, this theory is centred on the interactions amongst gene products from the offspring and mother, and predicts the expression of maternal alleles of imprinted genes to aid in this communication as these alleles are guaranteed to match the maternal genotype (Patten et al. 2014; Wolf and Hager 2006). In all land plants, there is a potential for interactions between the offspring genotype and maternal genotype (Fig. 2). As in all sexually reproducing organisms, the zygote interacts with any maternal factors deposited in the egg, though these factors would likely not persist for many cell divisions. Imprinting of genes expressed immediately after fertilization would be predicted, irrespective of whether this is in the embryo or endosperm. The fertilized egg is always initially surrounded by the maternal gametophyte, and in the case of bryophytes, the offspring remain in direct contact for the duration of its phase in the life cycle. In any case in which resources are transferred or growth is coordinated between mother an offspring, genes in the signalling pathway would be expected to be imprinted. Therefore, the prediction is for specific expression of maternal alleles to coordinate interactions between offspring and mother but does not allow for biased paternal expression.

In ANA-grade angiosperms, like in other angiosperms, the endosperm and embryo suspensor are likely to be the primary post-fertilization tissues involved in interacting with the maternal plant. The main difference of predictions made under the coadaptation hypothesis relative to the parental conflict or differential dosage hypotheses centres on the Nymphaeales and Austrobaileyales. Since the perisperm or nucellus are the main nutrient storage tissues for the developing embryo, a greater number of genes may be imprinted to better coordinate resource transfer to the embryo (Fig. 2C).

In ferns and lycophytes, the interaction between mother and embryo is often relatively brief, consisting of only the earliest stages of embryonic growth for ferns and lycophytes. While short, this interaction is at a crucial stage of the life cycle, thus imprinting of maternal genes to ensure proper coordination with the gametophytic mother of early growth could be expected to arise (Fig. 2B).

In bryophytes, the connection between mother and offspring is sustained and necessary (Fig. 2A). In the context of the coadaptation hypothesis, this strong relation between mother and offspring is expected to result in a large number of imprinted genes as many stages of development may need to be coordinated. Coordinated growth between embryos and mothers may favour imprinting of relevant genes in liverworts more strongly than in mosses and hornworts, as liverwort embryos spend a greater proportion of their life encapsulated by maternal tissue.

## Perspectives

To further our understanding of the evolution of imprinting in land plants, we propose three lines of investigation. In all cases, analyses of allele-specific gene expression from transcriptomes devoid of maternal RNA contamination is necessary. First, it is required to establish the presence or absence of imprinting in the endosperm and the embryo of ANA-grade angiosperms to elucidate whether imprinting in angiosperms is always prevalent in the endosperm. Second, investigating the expression of parental alleles in fern embryos will enable the hypothesis of whole chromosome imprinting to be revisited. Thirdly, predictions of imprinting in bryophytes need to be tested at the genomic level by sequencing parental allele specific transcriptomes.

To this end, several recently developed tools will aid in the investigation of imprinting in these species. Published genomes in Nymphaea (Povilus et al. 2020; Zhang et al. 2020b), ferns (Lang et al. 2018; Li et al. 2018, 2020; Marchant et al. 2019; Rensing et al. 2008; Zhang et al. 2020a) and bryophytes (Bowman et al. 2017; Carey et al. 2020a; McDaniel et al. 2007; Montgomery et al. 2020) provide good templates to sequence additional natural accessions required to establish parental allele specific transcriptomes. The recent utilization of single-cell RNA sequencing to uncover the spatial heterogeneity of imprinted gene expression in different functional domains of Arabidopsis endosperm (Picard et al. 2020) will provide interesting additional information regarding cell type specific imprinting and the function of imprinted genes. Single-cell RNA sequencing would be beneficial to use when looking at the reduced endosperm of ANA-grade angiosperms and early stages of embryogenesis in ferns and bryophytes, particularly focusing on the cells at the interface between embryos and mothers, because the cells in which imprinting may occur in these tissues may be a small percentage of the total population of cells in the tissue. Regardless of whether whole tissues or single cells are collected, special care must be taken to prevent contamination of transcriptomes by RNA from surrounding maternal tissues (Schon and Nodine 2017). Finally, assuming that parental genomic imprinting is found in ferns, lycophytes, and bryophytes, a bioinformatic comparison of imprinted genes across all land plant groups using a recently published pipeline (Picard and Gehring 2020) may help uncover if common pathways are affected.

### Author contribution statement

SAM and FB wrote the manuscript.
